# Evaluation of inflammatory and fibrosis biomarkers at different stages of degenerative mitral valve disease in dogs

**DOI:** 10.29374/2527-2179.bjvm005025

**Published:** 2025-08-18

**Authors:** Natalie Ruiz Algibay, M. Florencia Mosquillo, Ana Meikle, Alejandro Benech Gulla

**Affiliations:** 1 Unidad de Clínica y Cirugía de Pequeños Animales, Departamento de Clínicas y Hospital Veterinario, Facultad de Veterinaria, UDELAR, Montevideo, Uruguay.; 2 Unidad de Endocrinología y Metabolismo Animal (LEMA), Imagenología y Análisis Clínicos, Departamento de Clínicas y Hospital Veterinario, Facultad de Veterinaria, UDELAR, Montevideo, Uruguay

**Keywords:** veterinary cardiology, degenerative mitral valve disease, cardiac biomarkers, cardiologia veterinária, doença degenerativa da valva mitral, biomarcadores cardíacos

## Abstract

Degenerative mitral valve disease is the most prevalent heart disease in dogs. Research on biomarkers of heart diseases has increased recently owing to their value in providing complementary information to gold standard diagnostic methods and enhancing the understanding of pathophysiology. Novel biomarkers, such as Galectin-3 (Gal-3), soluble interleukin-1 receptor-like 1 protein (sST2), and growth differentiation factor 15 (GDF-15), have demonstrated prognostic value in human medicine but are poorly studied in veterinary medicine. The purpose of this study was to determine the serum concentrations of these novel inflammatory biomarkers, along with traditional biomarkers, in dogs at different stages of degenerative mitral valve disease. Thirty-eight dogs were included: 14 in stage A, 10 in stage B2, and 14 in stage C. Serum concentrations of five biomarkers (Gal-3, sST2, GDF-15, fibrinogen, and C-reactive protein), echocardiography, thoracic radiography, clinical chemistry, and blood cell counts were assessed for each dog. Differences in biomarker concentrations between groups were analyzed. Fibrinogen and C-reactive protein concentrations were higher in group C than in group A. Galectin-3 concentrations were higher in group B2 compared to those in group C. GDF 15 concentrations were higher in group B2 than in group A. No significant differences were found between groups B2 and C. sST2 concentrations did not differ between the groups. In conclusion, the novel inflammatory biomarker GDF-15 was measurable in dogs and was elevated in stage B2, similar to Gal-3, suggesting that inflammation and fibrosis begin with cardiac remodeling before clinical signs appear. Classical biomarkers showed the expected behavior. Further studies are needed to determine whether treatment affects the behavior of novel biomarkers.

## Resumo

A doença degenerativa da valva mitral é a doença cardíaca mais prevalente em cães. A pesquisa sobre biomarcadores em doenças cardíacas tem aumentado recentemente devido à sua utilidade, fornecendo informações complementares aos métodos diagnósticos padrão-ouro, além de colaborar na compreensão da fisiopatologia. Novos biomarcadores como Galectina-3, sST2 e GDF 15 demonstraram valor prognóstico na medicina humana, mas são pouco investigados na medicina veterinária. O objetivo deste estudo foi determinar as concentrações séricas desses novos biomarcadores inflamatórios e de outros já existentes em cães com diferentes estágios de doença degenerativa da valva mitral. Trinta e oito caninos foram incluídos, quatorze no estágio A, dez no estágio B2 e quatorze no estágio C. Foram realizadas análises das concentrações séricas de cinco biomarcadores (Galectina-3, sST2, GDF-15, fibrinogênio e proteína C reativa), ecocardiografia, radiografia de tórax, bioquímica clínica e hemograma em cada cão. As diferenças nas concentrações dos biomarcadores entre todos os grupos foram investigadas. As concentrações de fibrinogênio e proteína C reativa no grupo C foram maiores que no A. Galectina-3 foi maior no grupo B2 em comparação ao C. GDF 15 foi maior no grupo B2 do que no A. Não foram encontradas diferenças entre os grupos B2 e C. As concentrações de sST2 não mostraram diferenças entre nenhum grupo. Em conclusão, o novo biomarcador de inflamação GDF-15 é mensurável em caninos e foi maior no estágio B2, semelhante à galectina-3, o que pode significar que a inflamação e a fibrose começam com a remodelação cardíaca, antes do aparecimento dos sinais clínicos. Os biomarcadores clássicos apresentaram o comportamento esperado. Mais estudos são necessários para determinar se o tratamento tem efeito no comportamento dos novos biomarcadores.

## Introduction

Degenerative mitral valve disease (DMVD) is the most prevalent heart disease in canines (Atkins et al., 2009; Borgarelli & Häggström, 2010). Although this disease primarily affects the heart, it triggers multiple systemic repercussions that can only be assessed when complementary studies are performed (Domanjko Petrič et al., 2018; Nicolle et al., 2007; Pouchelon et al., 2015; Tarnow et al., 2007; Yu & Huang, 2016). Degenerative mitral valve disease is a chronic disease with a relatively benign course, and its asymptomatic phase can persist for several years. However, this group can be heterogeneous, with canines presenting moderate valvular deterioration and others on the verge of developing heart failure (HF) (Borgarelli & Häggström, 2010). Once the clinical phase is reached, the prognosis can also vary (Borgarelli et al., 2008; Häggström et al., 2008).

Degenerative mitral valve disease has been traditionally considered a non-inflammatory disease because there is no infiltration of inflammatory cells into the mitral leaflets or myocardium (Buchanan, 1977). However, more recent studies have demonstrated the expression of cytokines in the atrial and ventricular myocardium in different diseases that cause HF, including DMVD (Fonfara et al., 2013). Furthermore, increased expression of genes related to inflammatory processes has been observed in canines in the most advanced phases of DMVD (Oyama & Chittur, 2006). In human medicine, it has been widely reported that systemic inflammation plays a major role in the pathophysiology and outcome of congestive heart failure (CHF), as well as in local cardiac remodeling (Rauchhaus et al., 2000). Biomarkers have gained prominence due to their proven usefulness in patients with CHF (Ayça et al., 2015).

Standard biomarkers of systemic inflammation include C-reactive protein (CRP) and fibrinogen. There is evidence of increased levels of these markers in advanced stages of DMVD in dogs (Cunningham et al., 2012; Polizopoulou et al., 2015; Reimann et al., 2016). In human medicine, new evidence supports the use of Galectin-3 (Gal-3), soluble interleukin-1 receptor-like 1 protein (sST2) (Bayes-Genis et al., 2014; Emdin et al., 2018), and growth differentiation factor 15 (GDF-15) (Sharma et al., 2017) as predictors of outcomes and mortality in patients with various chronic inflammatory diseases, including HF. These biomarkers are not specific to a particular pathology but are general indicators of disease severity and mortality (Du et al., 2018). Their prognostic value has been demonstrated in patients with HF (Meijers et al., 2021). Moreover, the latest guidelines from the American College of Cardiology and the American Heart Association for the management of HF include sST2 and Gal-3 as biomarkers for stratifying patients with acute and chronic HF (Yancy et al., 2017). However, these biomarkers have been poorly studied in veterinary medicine (Klein et al., 2022).

This study aimed to jointly evaluate traditional biomarkers (CRP and fibrinogen) and novel biomarkers (Gal-3, sST2, and GDF-15) associated with systemic inflammation and its consequences on cardiac tissue, including fibrosis, in canines at different stages of DMVD.

## Material and methods

This prospective study was approved by the Ethics Committee of the Facultad de Veterinaria, UDELAR, and was conducted at the Teaching Veterinary Hospital. Forty-seven client-owned canines were initially selected according to the American College of Veterinary Internal Medicine recommendations. The animals were classified as having DMVD stage A, DMVD subclinical stage B, or DMVD clinical stage C (Keene et al., 2019). All dogs underwent clinical evaluation and paraclinical studies, as detailed below. Blood samples were drawn from the cephalic vein using 21G needles. The samples were placed in serum, citrate, and EDTA tubes. Blood samples for biomarker analysis were centrifuged for 15 minutes at 1500 g at room temperature. The supernatant (serum or plasma) was then aliquoted into Eppendorf tubes and stored at −80 °C within 60 minutes of sample collection.

### Exclusion criteria

Only dogs with a normal complete blood count and normal serum biochemistry, according to Oregon State University reference values, were recruited to exclude animals with possible non-cardiac inflammatory or systemic diseases. Any dogs with anemia, leukocytosis, leukopenia, neutrophilia, eosinophilia, abnormal levels of albumin or total proteins, elevated liver enzymes (alanine aminotransferase and aspartate aminotransferase), cholesterol, or renal biomarkers (urea and creatinine) were excluded from the study.

### Clinical cardiological evaluation

Complete clinical and cardiologic evaluations were performed for each dog, including assessment of mucous membrane coloration, capillary refill time, heart and respiratory rate, pulse quality, and cardiac and pulmonary auscultation. Heart murmurs were classified according to an intensity scale (1 to 6) (Kvart & Häggström, 2002). Systolic blood pressure was measured using an oscillometric device (Petmap, USA), following the methodology outlined by Acierno et al. (2018).

### Laboratory analyses

#### Hematology

Complete blood counts were performed using a Mythic 18 Vet automated analyzer (Orphée, Geneva, Switzerland), with reagents and controls sourced from the same supplier. May–Grünwald Giemsa staining was subsequently used to evaluate blood smears under a Nikon Eclipse E100 optical microscope.

#### Biochemistry

Serum biochemical analyses were performed using a CB350i automated analyzer (Wiener Lab Group, Rosario, Argentina), with all reagents and controls obtained from the same supplier. The following parameters were measured: glycemia, triglycerides, alanine aminotransferase, aspartate aminotransferase, serum alkaline phosphatase, total protein, albumin, globulin, total cholesterol, HDL cholesterol, LDL cholesterol, total bilirubin, urea, and creatinine.

Coagulation tests included prothrombin time, activated partial thromboplastin time, and fibrinogen levels, and were measured using a Cor 50 analyzer (Wiener Lab Group, Rosario, Argentina). For all metabolites measured, the coefficients of variation for the controls were below 10%.

#### Radiography

Thoracic radiographs were taken in two projections (laterolateral and dorsoventral) using a Vetter Rems device (Vetter GmbH, Tuttlingen, Germany). Tranquilizing drugs were not administered. All visible thoracic structures were evaluated, with particular attention to the heart and lungs, to assess for the presence of pulmonary edema. Cardiac size was assessed using the vertebral heart score (VHS) (Buchanan & Bücheler, 1995), and left atrial enlargement was evaluated using the vertebral left atrial size (VLAS) scale (Malcolm et al., 2018).

#### Echocardiography

To diagnose DMVD and determine disease stage, echocardiographic examinations were performed using a Toshiba Nemio device (Japan) with 3.5–7.5 MHz phased-array transducers. The echocardiographic protocol followed the methods described by Thomas (1984) and Boon (2011).

In B-mode, the aortic annulus (Ao) and left atrial (LA) cavity were measured using a transverse section of the cardiac base, and the LA/Ao ratio was calculated to assess left atrial enlargement. In M-mode, measurements included left ventricular (LV) cavity dimensions, interventricular septal thickness, and left ventricular free wall thickness in both systole and diastole. These measurements were obtained from a transverse section of the LV at the level of the chordae tendineae. Each parameter was measured three times and averaged. The left ventricular shortening fraction (LVFS) was calculated from systolic and diastolic LV dimensions (Boon, 2011). All values were normalized to body weight according to the method described by Cornell et al. (2004).

Simultaneous electrocardiography was performed using leads I, II, III, aVR, aVL, and aVF with a Fukuda Denshi device (Japan). Electrocardiographic interpretation was based on lead II, following the recommendations of Tilley et al. (2025).

#### Biomarkers

Serum concentrations of CRP, Gal-3, sST2, and GDF-15 were determined using commercial enzyme-linked immunosorbent assay (ELISA) kits, following the manufacturers’ instructions. C-reactive protein levels were measured using a Tridelta Phase Range CRP Canine Assay ELISA kit (Tridelta Development Ltd., County Wicklow, Ireland). Galectin-3 was measured using a Canine GAL3 ELISA Kit (MBS740224, MyBioSource, San Diego, CA, USA); sST2 was measured using a Canine Interleukin-33 Receptor (ST2) ELISA Kit (MBS9346824, MyBioSource); and GDF-15 was measured using a Canine GDF15 ELISA Kit (MBS2603755, MyBioSource). Optical density values were obtained using a Multiskan™ FC Microplate Photometer (Thermo Scientific, Waltham, MA, USA), and analyte concentrations were calculated from standard curves.

#### Statistical analyses

Data were analyzed using the Statistical Analysis System (SAS® OnDemand for Academics). Normality was assessed using the PROC UNIVARIATE procedure. Non-normally distributed data were log-transformed prior to analysis. A mixed model procedure was used, with DMVD stage (A, B, or C) included as a fixed effect. Differences were considered statistically significant at *p <* 0.05, while *p*-values between 0.05 and 0.10 were interpreted as showing a tendency.

## Results

Only animals with normal complete blood counts and biochemical parameters, based on Oregon State University reference values, were included in the study. Accordingly, three animals in group B2, one in group C, and five in group A were excluded. This exclusion criterion was applied to minimize the influence of other diseases on biomarker concentrations.

Thirty-eight dogs met the inclusion criteria: DMVD stage A (n = 14), DMVD stage B2 (n = 10), and DMVD clinical stage C (n = 14). The mean age in group A was 8.6 years (range: 7–12 years), in group B2 was 11.6 years (range: 7–15 years), and in group C was 11.9 years (range: 9–15 years). The sex distribution (male and female) was similar across all groups.

Group A included eight mixed-breed dogs, four Poodles, one Dachshund, and one Yorkshire Terrier. Group B2 included four mixed-breed dogs, three Poodles, two Shih Tzus, one Dachshund, and one Yorkshire Terrier. Group C consisted of five Poodles, four mixed-breed dogs, three Yorkshire Terriers, one Shih Tzu, and one Dachshund (Table 1).

**Table 1:** Comparison of selected clinical, echocardiographic, radiographic, and electrocardiographic parameters in dogs at different stages of degenerative mitral valve disease (mean ± standard deviation).

Parameter	Stage A	Stage B2	Stage C
	(n = 14)	(n = 10)	(n = 14)
*Demographics*			
Body weight (kg)	10.1 ± 1.0^a^	9.1 ± 1.1^abx^	6.1 ± 1.0^by^
Sex (F/M)	8/6	5/5	7/7
Age (years)	8.6 ± 0.6^a^	11.6 ± 0.7^b^	11.9 ± 0.6^b^
*Clinical cardiology*			
Heart Rate (bpm)	127.1 ± 5.1^a^	144.0 ± 6.1^b^	150.0 ± 5.1^b^
Murmur (1-6)	0 ± 0.1^a^	2.5 ± 0.1^b^	3.9 ± 0.1^c^
*Echocardiography*			
LA n	0.84 ± 0.08^x^	0.95 ± 0.09^xy^	1.07 ± 0.08^y^
LA/Ao	1.16 ± 0.05 ^a^	1.60 ± 0.05 ^b^	1.82 ± 0.05 ^c^
LVIDd n	1.46 ± 0.08^a^	1.53 ± 0.09^abx^	1.75 ± 0.08^by^
LVFS (%)	0.40 ± 0.02^a^	0.44 ± 0.02^a^	0.50 ± 0.02^b^
*Radiography*			
VHS (cv)	10.5 ± 0.2^a^	10.7 ± 0.2ª	11.3 ± 0.2^b^
VLAS (cv)	1.9 ± 0.08ª	2.3 ± 0.1^b^	2.6 ± 0.8^c^
*Electrocardiography*			
P wave (s)	0.035 ± 0.001^a^	0.038 ± 0.02^a^	0.046 ± 0.001^b^
QRS duration (s)	0.052 ± 0.002^a^	0.053 ± 0.002^a^	0.060 ± 0.001^b^
QRS amplitude (mV)	1.49 ± 0.13^x^	1.81 ± 0.15^xy^	1.82 ± 0.13^y^

*LA n:* normalized left atrial diameter; *LA/Ao:* left atrium-to-aortic root ratio; *LVIDd n:* normalized left ventricular internal diastolic diameter; *LVFS:* left ventricular shortening fraction; *VHS:* vertebral heart score; *VLAS:* vertebral left atrial size. a, b, c indicate statistically significant differences between groups (p < 0.05); x, y indicate tendencies toward significance (p < 0.1).

Heart rate varied according to disease stage, being lower in group A than in groups B2 and C (*p* = 0.01); however, there was no significant difference between groups B2 and C. The intensity of the heart murmur increased progressively with disease stage (*p* < 0.001) (Table 1).

### Echocardiographic, radiographic, electrocardiographic, and laboratory findings

Radiographic VHS was higher in group C than in groups A (*p* = 0.01) and B2 (*p* = 0.01), with no significant difference between groups A and B2. The VLAS scale showed statistically significant differences between all groups, increasing progressively with disease stage (*p* < 0.001) (Table 1).

In the echocardiographic evaluation, the LA/Ao ratio differed between all groups, increasing with disease severity (*p* < 0.001), as expected based on the inclusion criteria. Most M-mode echocardiographic measurements, normalized to body weight, showed no statistically significant differences. However, two parameters used as inclusion criteria were affected. The normalized left atrial diameter (LAn) tended to be larger in group C than in group A (*p* = 0.052). The normalized left ventricular internal diameter in diastole (LVIDd-n) was significantly greater in group C than in group A (*p* = 0.026), with a tendency toward a greater value in group C compared to group B2 (*p* = 0.07). A group effect was also observed for LVFS, which was significantly higher in group C (*p* < 0.001) compared to groups A and B2 (Table 1).

Electrocardiographic evaluation revealed sinus rhythm in all groups. P wave duration was significantly longer in group C than in group A (*p* < 0.001), with no differences between groups A and B2. QRS complex duration also differed by group, with group C showing higher values than groups A and B2 (*p* = 0.01) (Table 1). No other electrocardiographic parameters showed significant changes.

In the biochemical analysis, only urea and creatinine concentrations were significantly affected by disease stage. Urea levels were approximately twice as high in group C compared to groups A and B2 (*p* = 0.006), with no significant difference between groups A and B2. Creatinine concentrations were also higher in group C than in groups A and B2 (*p* = 0.038), with no significant difference between groups A and B2.

Complete blood count and biochemical parameters used for exclusion criteria are presented in Table 2.

**Table 2:** Comparison of selected biochemical parameters and complete blood count in dogs at different stages of degenerative mitral valve disease (mean ± standard deviation).

Parameter	Stage A	Stage B2	Stage C
	(n = 14)	(n = 10)	(n = 14)
*Biochemistry*			
Albumin (g/dL)	3.5 ± 0.05	3.4 ± 0.06	3.5 ± 0.05
Total proteins (g/dL)	6.2 ± 0.1	6.5 ± 0.2	6.4 ± 0.1
ALT (U/L)	33.9 ± 5.7	24.2 ± 6.8	42.7 ± 5.7
AST (U/L)	58.4 ± 10.4	59.9 ± 12.3	78.2 ± 10.4
Cholesterol (mg/dL)	209.4 ± 12.5	195.0 ± 14.8	187.6 ± 15.0
Urea (mg/dL)	34.0 ± 11.2^a^	40.6 ± 13.2^a^	84.5 ± 11.2^b^
Creatinine (mg/dL)	0.74 ± 0.09^ax^	0.81 ± 0.10^aby^	1.08 ± 0.09^b^
*Complete blood count*			
Erythrocytes (10^6^/µL)	7.8 ± 0.3	7.5 ± 0.4	7.7 ± 0.3
Hemoglobin (g/dL)	18.1 ± 0.7	17.2 ± 0.8	17.6 ± 0.7
Hematocrit (%)	43.9 ± 2.0	43.8 ± 2.4	44.8 ± 2.1
Leukocytes (/mm^3^)	11471 ± 786	9910 ± 930	10177 ± 816
Lymphocytes (/mm^3^)	2413.3 ± 291.8	2104.9 ± 345.2	2024 ± 302.8
Neutrophils (/mm^3^)	7650.8 ± 626.8	6879.9 ± 741.6	6821 ± 650.4
Monocytes (/µL)	419.6 ± 90.1	266.10 ± 106.4	398.2 ± 93.3
Eosinophils (/µL)	655.4 ± 103.0	551.7 ± 121.8	552.5 ± 106.8

*ALT*: alanine aminotransferase; *AST*: aspartate aminotransferase. a, b, c indicate statistically significant differences between groups (p < 0.05); x, y indicate tendencies toward significance (p < 0.1).

### Biomarkers

The health status of the animals was influenced by fibrinogen, CRP, Gal-3, and GDF-15 concentrations. Fibrinogen concentrations in group C were significantly higher than those in group A (*p* = 0.012) and tended to be higher than those in group B2 (*p* = 0.085). No significant differences were observed between groups B2 and C. C-reactive protein levels in group C were significantly higher than in group A (*p* = 0.0001) and group B2 (*p* = 0.031). No differences were found between groups A and B2. Galectin-3 concentrations were significantly higher in group B2 than in group C (*p* = 0.014) and tended to be higher than in group A (*p* = 0.052). No significant differences were found between groups A and C. GDF-15 levels were significantly higher in group B2 than in group A (*p* = 0.012) and tended to be higher in group C compared to group A (*p* = 0.087). No differences were observed between groups B2 and C. sST2 concentrations did not differ significantly between any of the groups (Table 3).

**Table 3:** Serum biomarker concentrations in dogs at different stages of degenerative mitral valve disease (mean ± standard deviation).

Biomarker	Stage A	Stage B2	Stage C
Gal-3 (ng/mL)	3.29 ± 0.41^ax^	4.23 ± 0.48^aby^	2.64 ± 0.41^a^
sST2 (ng/mL)	2.75 ± 0.41	2.81 ± 0.48	2.90 ± 0.41
GDF-15 (pg/mL)	183.52 ± 42.35^ax^	378.40 ± 50.11^b^	288.63 ± 42.35^by^
CRP (µg/mL)	1.38 ± 0.72^a^	2.29 ± 0.85^a^	5.55 ± 0.72^b^
Fibrinogen (mg/dL)	217.36 ± 71.52^ax^	280.10 ± 32.56^aby^	322.50 ± 27.52^b^

Gal-3, Galectin 3; sST2, soluble interleukin-1 receptor-like 1 protein; GDF-15, growth differentiation factor 15; CRP, C-reactive protein. a, b, c indicate statistically significant differences between groups (p < 0.05); x, y indicate tendencies toward significance (p < 0.1).

## Discussion

Chronic inflammation and fibrosis play a major role in the pathogenesis and progression of CHF. Inflammatory processes contribute to cardiac depression and accelerate disease progression. Cardiac fibrosis impairs ventricular function, leading to both systolic and diastolic dysfunction, thereby promoting the advancement of heart failure. These pathological changes are mediated by inflammatory molecules that act on cardiac myocytes and fibroblasts (Savic-Radojevic et al., 2017). The association between CRP levels and heart failure was first reported in 1956 (Elster et al., 1956). Since then, inflammatory biomarkers have been extensively studied in cardiology.

Fibrinogen is an acute-phase protein whose production increases in response to inflammation (Torrente et al., 2015). It is classified as a minor acute-phase protein, as its concentration increases 2–4 times above baseline and remains elevated for prolonged periods (Kjelgaard-Hansen & Jacobsen, 2011). Recent studies in human medicine have renewed interest in fibrinogen as a cardiovascular biomarker. Pieters et al. (2021) confirmed that fibrinogen not only contributes to the development of cardiovascular diseases but also influences disease progression and outcomes. Limited studies have investigated fibrinogen concentrations in canines with heart disease, including DMVD. However, available reports show significantly elevated levels in dogs with heart failure compared to healthy controls (Prihirunkit et al., 2014; Tarnow et al., 2004; Tarnow et al., 2007), which is consistent with the findings of this study.

C-reactive protein is a major acute-phase protein, and its concentration can increase 100- to 1,000-fold in response to inflammation (Eckersall & Bell, 2010). In our study, CRP concentrations were significantly higher in the DMVD group C compared to groups A and B2. However, this did not allow for discrimination between groups A and B2, consistent with findings by Rush et al. (2006), Reimann et al. (2016), and Domanjko Petrič et al. (2018). Cunningham et al. (2012) also reported increased CRP levels in dogs with CHF due to DMVD or dilated cardiomyopathy, which were associated with disease severity.

In the present study, two dogs showed the highest CRP values (12 µg/mL). These individuals may have been further along in disease progression, as both had remained in stage C for approximately 48 months, which is substantially longer than the other dogs in the same group. Ljungvall et al. (2010) did not find an association between CRP levels and disease severity, although, similarly to our findings, they reported values above 10 µg/mL in 4 of 18 dogs classified as stage C. Older dogs may exhibit elevated CRP levels due to age-related conditions such as periodontitis or osteoarthritis, which can independently raise systemic inflammation markers (Hurter et al., 2005). However, in our study, dogs in groups B2 and C were of similar age, yet CRP levels were significantly higher in group C.

In human medicine, elevated CRP levels during HF are well documented, as tissue necrosis is a major trigger for CRP release and serves as a key prognostic factor in acute myocardial infarction (Hirschfield & Pepys, 2003). While acute myocardial infarction is uncommon in dogs, frequent histological findings in chronic DMVD include microscopic intramural myocardial infarcts, myocardial arteriosclerosis, and myocardial fibrosis (Falk et al., 2006). Arteriosclerosis may impair vasodilatory capacity and, when combined with increased left ventricular wall stress from volume overload due to mitral regurgitation, can result in insufficient oxygen delivery. This insufficiency may lead to cardiomyocyte death and contribute to elevated CRP levels (Falk et al., 2010).

Galectins are important regulators of inflammatory and immune responses and are expressed in many types of inflammatory cells (Zaborska et al., 2023). Galectin-3 is predominantly released during the differentiation of monocytes into macrophages and plays a role in multiple processes of the acute inflammatory response, including neutrophil activation and adhesion, monocyte chemoattraction, opsonization of apoptotic neutrophils, and mast cell activation (Henderson & Sethi, 2009). Gal-3 has been shown to be involved in inflammation, fibrosis, cell–matrix interactions, cell proliferation, and protection against apoptosis (Sygitowicz et al., 2021). Lee et al., 2022 evaluated serum Gal-3 concentrations in healthy dogs, dogs with heart disease, and dogs with non-cardiac diseases (e.g., endocrine or neoplastic). They found higher concentrations of Gal-3 in dogs with cardiac (1.12 ± 0.83 ng/mL) and non-cardiac diseases (2.27 ± 2.59 ng/mL) compared to healthy dogs (0.64 ± 0.15 ng/mL). However, when analyzing only the subgroup with DMVD, no significant differences were found compared to the healthy group, which is in contrast with the findings of our study. It is important to note that the mean Gal-3 concentration in our group A (which corresponds to the "healthy" group in Lee et al.) was considerably higher (3.29 ± 0.41 ng/mL). One possible explanation is that Lee et al.’s healthy group consisted entirely of young Beagle dogs (mean age: 2.38 ± 0.52 years). There is evidence in both humans (de Boer et al., 2012; Seropian et al., 2023) and dogs (Arcuri et al., 2024) of a positive correlation between Gal-3 concentrations and age. Arcuri et al. (2024) studied Gal-3 levels in dogs with DMVD, with and without atrial enlargement, and compared them to healthy dogs. Their healthy group had similar Gal-3 concentrations (3.9 ± 1.65 ng/mL) to those in our study, with a slightly higher median age (10 ± 3 years). Consistent with our findings, Lee et al., 2022 also reported that dogs with stage B2 DMVD had higher Gal-3 concentrations (1.05 ± 0.90 ng/mL) than those with stage C (0.80 ± 0.36 ng/mL), although the difference was not statistically significant. As in our study, there was no significant age difference between the B2 and C groups. Sakarin et al. (2016) reported significantly higher serum Gal-3 concentrations in dogs with DMVD compared to healthy controls, but no differences among disease stages. They also detected significantly increased Gal-3 levels in cardiac tissue from DMVD dogs compared to healthy dogs. The treatment status of the DMVD dogs was not specified. Two other clinical studies reported similar findings. Rešetar Maslov et al. (2023) administered pimobendan and furosemide to dogs at all DMVD stages. Kim et al. (2023) did not provide detailed treatment data. In their study, five of ten dogs in stage B2 received pimobendan, while in stage C, most dogs received a combination of pimobendan, enalapril, benazepril, spironolactone (10/14), and/or furosemide (5/14). Galectin-3 plays a key role in tissue fibrosis and ventricular remodeling (Zaborska et al., 2023). While inflammation is necessary for tissue repair, sustained inflammation can lead to tissue damage and organ dysfunction. Gal-3 provides cardioprotective effects through anti-apoptotic and anti-necrotic mechanisms. However, prolonged elevation of Gal-3 promotes the release of mediators that stimulate cardiac fibroblast proliferation, collagen synthesis, extracellular matrix deposition, and ultimately, ventricular dysfunction (Savic-Radojevic et al., 2017). Angiotensin-converting enzyme inhibitors (ACEIs) and mineralocorticoid receptor antagonists (MRAs) may reduce pathological cardiac remodeling. Chronic aldosterone excess is associated with increased oxidative stress and myocardial inflammation (Ames et al., 2017). Both classes of drugs were used in group C dogs in our study and could explain the lower Gal-3 concentrations observed in this group compared to group B2. However, results from prior studies on the effects of these drugs on Gal-3 levels are inconsistent. In human clinical trials, MRAs have not consistently altered Gal-3 concentrations in patients with chronic HF due to left ventricular systolic dysfunction (Gandhi et al., 2015). In contrast, a recent study by Xie et al. (2024) reported a reduction in several biomarkers, including Gal-3, in HF patients treated with spironolactone. Experimental models also suggest that MRAs favorably modulate local Gal-3 expression in myocardial tissue, although serum levels do not always correlate with myocardial expression (Lax et al., 2015). In a murine model of fibromyxomatous degeneration of the mitral valve, spironolactone attenuated both interstitial and perivascular fibrosis and inhibited myocardial Gal-3 expression (Ibarrola et al., 2020). In dogs, Klein et al. (2022) found no significant differences in Gal-3 serum concentrations between healthy animals and those with DMVD, regardless of disease stage. Moreover, no changes were observed over a six-month follow-up period, even in dogs receiving treatment. Almost all dogs in B2 and C stages were on medication at the study’s outset, although only a few received ACEIs or MRAs. In human medicine, Gal-3 is considered a biomarker of cardiac remodeling and an indicator of adverse cardiac events, including risk of CHF onset and myocardial fibrosis (Milting et al., 2008). The magnitude of Gal-3 elevation reported in humans with CHF is generally higher than in dogs with DMVD. This discrepancy may reflect species differences or differences in disease pathophysiology. Human cardiovascular diseases such as ischemic heart disease, hypertension, and cardiomyopathies typically lead to more extensive cardiac fibrosis than DMVD in dogs (Sakarin et al., 2016).

There are two isoforms of ST2: transmembrane and soluble. The transmembrane isoform plays an immunomodulatory role through signaling mediated by interleukin-33 (IL-33). IL-33 secretion activates downstream signaling pathways, thereby preventing cardiomyocyte hypertrophy. In contrast, the soluble isoform of ST2 (sST2) acts as a decoy receptor for IL-33, attenuating its cardioprotective effects. In human medicine, elevated sST2 concentrations have been observed in cancer and inflammatory conditions, including HF (Savic-Radojevic et al., 2017). sST2 is recognized as a novel biomarker of HF and has been included in the American College of Cardiology and the American Heart Association guidelines (2013) for risk stratification in patients with acute and chronic HF. In veterinary medicine, there are limited studies on the behavior of sST2 in dogs with DMVD. The serum sST2 values reported in our study were similar to those previously described by Klein et al. (2022). In line with our findings, both Klein et al. (2022) and Rešetar Maslov et al. (2023) reported no significant differences in sST2 concentrations between healthy controls and dogs with DMVD, nor among different disease stages.

Growth differentiation factor-15 is an anti-inflammatory cytokine that is typically undetectable in peripheral blood under physiological conditions. Its expression is markedly upregulated in cardiomyocytes following ischemia/reperfusion injury, particularly in the infarct border zone, where it may exert cardioprotective effects (Savic-Radojevic et al., 2017). In patients with chronic HF, elevated GDF-15 concentrations have been associated with disease severity and are considered to have prognostic value (Kosum et al., 2024). Sabbah et al. (2016) reported plasma GDF-15 concentrations in an experimental model of HF in dogs with microembolization-induced cardiac injury. These dogs exhibited significantly higher GDF-15 levels than those observed in our study (314 ± 29 pg/mL at baseline and 875 ± 71 pg/mL following the induction of HF). To our knowledge, this is the first report describing serum GDF-15 levels in dogs with naturally occurring DMVD. In our study, higher concentrations were found in groups B2 and C compared to group A, with no significant differences between the DMVD stages. These findings suggest that GDF-15 levels are increased in dogs with DMVD, regardless of clinical status. In both healthy humans and patients with HF, GDF-15 levels increase with age (Teramoto et al., 2024; Welsh et al., 2022). This age-related trend may also be reflected in our results, as dogs in group A were younger than those in groups B2 and C, whose ages were comparable. Although group C represents the clinical stage of DMVD, GDF-15 concentrations were slightly lower than in group B2. In human patients with acute or decompensated chronic HF, a significant decline in GDF-15 levels during hospitalization has been associated with better clinical outcomes, including a reduced risk of 30-day rehospitalization (Kosum et al., 2024). These reductions are likely treatment-related. There is evidence that angiotensin receptor blockers can modulate GDF-15 expression (Frank et al., 2008). In patients with severe non-ischemic dilated cardiomyopathy, GDF-15 concentrations in myocardial tissue—assessed during surgical procedures—were strongly correlated with the severity of myocardial fibrosis. Notably, one month after surgical intervention, GDF-15 levels significantly decreased, reinforcing its association with myocardial dysfunction severity (Lok et al., 2012).

### Limitations

Although all dogs underwent clinical and paraclinical evaluations, completely excluding non-cardiac inflammatory conditions remained challenging. Ongoing medical treatments may influence biomarker concentrations; therefore, future studies in untreated dogs are needed to clarify the effects of therapy on biomarker expression. Age is another factor that may affect biomarker levels. Group A, which served as the control group, was younger than groups B2 and C, which were of similar age. Identifying older dogs without comorbid conditions that fit the criteria for Group A proved difficult. Nonetheless, the study design allowed for consideration of this limitation in the interpretation of results. Additionally, incorporating established cardiac biomarkers such as NT-proBNP and troponin I, alongside novel markers like fibrinogen and CRP, would provide a more comprehensive assessment and enhance future research.

## Conclusions

In conclusion, the novel inflammatory biomarker GDF-15 was detectable in dogs and showed higher concentrations in stage B2, similar to Gal-3, suggesting that inflammation and fibrosis begin during cardiac remodeling, prior to the onset of clinical signs. Classical biomarkers behaved as expected. Further studies are needed to evaluate whether medical treatment influences the expression or diagnostic utility of these novel biomarkers.
